# Variable and fixed rate debt: Gambling on the future state of the economy after Covid-19

**DOI:** 10.1007/s11187-026-01230-7

**Published:** 2026-05-22

**Authors:** Marc Cowling, Kit Pong Wong, Huan Yang

**Affiliations:** 1 Oxford Brookes Business School, Oxford, UK; 2 University of Hong Kong, Hong Kong, China

**Keywords:** Variable rate loans, Fixed rate loans, Covid-19, Interest rate insurance, G21, G40, D24, D25, D53

## Abstract

After a decade of low and stable interest rates, recent global events have introduced a significant degree of economic turbulence causing a spike in inflation and central banks across the world to raise their base rates. This in turn was transmitted to commercial lenders who raised their loan interest rates. In this paper we use a large UK firm survey for 2020–2023 to consider the extent to which firms were able to access fixed or variable rate loans, and the types of firms and debt associated with each type of loan interest rate. This is important as fixed rate loans insure the borrower against future adverse states of the economy whilst lenders may prefer variable rate loans as this insures them against future volatility and uncertainty, and shifts risk to the borrower. Our key findings are that only 19.5% of loans were issued at a variable rate and that larger and more risk-tolerant firms received variable rate loans. We also found that mortgage debt was the most likely to be issued at variable rates of interest.

## Abstract (plain language summary)

Being stuck with variable rate debt when inflation and interest rates are increasing puts many firms in a difficult position as their costs of servicing outstanding debt put a cash strain on their businesses. It follows that the initial choice of what type of debt to use is of critical importance as not all types of debt have the same probability of being issued on a variable rate of interest. Our findings show that variable interest rates are most prevalent on mortgage debt, bank loans and overdrafts and least prevalent on P2P lending. We also find that firms that are more willing to take investment risks tend to be offered variable rate debt. Thus, firms need to carefully consider what their attitude to risk is and what types of debt they should seek if they anticipate that the future state of the economy will deteriorate.

## Introduction

As many latent and actual borrowers have found out over the last three years, loans have become harder to access, and the interest rates charged on loans have risen significantly. This has been a common feature across personal lending, mortgage lending, and commercial lending markets. In the UK alone, more than 800,000 mortgage holders saw the end of their fixed interest rate loan period in 2023 and were switched onto the standard variable rate^1^ which has caused a significant increase in repayment costs and mortgage default. This occurred at a period in time after the Covid-19 pandemic that saw UK inflation increase from 5.1% in November 2021 to 11.1% in October 2022 and Bank of England base rates rise to 0.25% on 15th of December 2021, up from 0.1%, and following a further thirteen base rate increases reaching a peak of 5.25% on 21 st July 2023.

This must be set within the wider and important context of small business access to finance, the terms and conditions under which debt is offered to them, and how the wider institutional context impacts both, all of which lead us to focus on small firms’ financing. For example, Beck and Demirguc-Kunt (2006) emphasise the major contribution of small firms to economic growth, but stress their relative growth constraints due to access to finance issues, whilst Block et al. (2018) argue that the landscape for entrepreneurial finance has changed dramatically recently and that these changes may improve small firms’ access to finance going forward. Fare et al. (2024) and Dejardin et al. (2024), in different contexts, found that bankruptcy law was fundamental in understanding smaller firms’ access to finance through its impact on the supply-side of debt markets. Cowling and Sclip (2023) found that these finance problems can often lead small firms to self-exclude from debt markets as they perceive that they will receive an unfavourable outcome which then disadvantages the firm who is investment constrained. The knowledge gap we fill is relatively unexplored but directly connects to this wider body of knowledge on access to finance problems that small firms face and the terms on which debt finance is offered when it is available.

For small firms in particular, the unanticipated disruption to their activities from the Covid-19 pandemic was severe and required unprecedented public intervention in the loan market to support their survival (Calabrese et al., 2022; Chundakkadan et al., 2022; Goodhart et al., 2023). What followed in terms of inflation and central banks’ base rates added to their already precarious financial state. However, it is also the case that some firms who raised loans during the pandemic and post-pandemic period were contractually insured against future ‘bad’ states of the economy as they had fixed rate loans which transferred risk from the borrower to the lender (Cowling & Wong, 2025). The conventional wisdom is that a borrower opts for the fixed rate loan if interest rates are anticipated to rise in the future or opts for the variable rate loan if interest rates are anticipated to fall (Carmichael & Handford, 2015). Equally, if the economy had returned to a ‘good’ state, then fixed rate borrowers would have been at a relative disadvantage as variable interest lending rates declined.

On the other hand, firms with variable rate loans faced the dramatic increases in loan interest rates that mirrored the central bank rate increases since 15th December 2021 in the UK. Thus, variable rate lenders had shifted risk to their borrowers in what turned out to be a ‘bad’ state of the economy. In some cases, this directly resulted in firms repaying their outstanding Covid-19 debt ahead of schedule (Cowling & Wilson, 2023). This is a completely different economic climate to the pre-Covid-19 period when the prevailing low-interest-rate landscape led to a decision whether to secure these low long-term interest rates with a fixed rate agreement or to benefit from the current negative short-term rates with a variable rate loan (Fischer & Kampl, 2019), and when the historical volatility of interest rates was low (Albertazzi et al., 2024).

It is perhaps surprising that we know quite a lot about what interest rates firms, and particularly SMEs, pay on their loans, but know rather less about the precise nature of the interest rates applied. On the former, extensive research has shown that SMEs faced increases in the price of loans during the global financial crisis period (Drakos, 2013), that small firms face a loan price premium due to a lack of market power (Dietrich, 2012), and that there is an interest rate reducing certification effect from big 4 auditors (Tsai & Hua, 2009). In addition, spatial loan markets appear to be commonplace and this results in interest rate penalties for firms in peripheral regions (Cowling et al., 2020) and lower interest rates for firms located in the most economically and technologically advanced regions (Ughetto et al., 2017), as well as a more general firm-bank distance effect (Bellucci, Borisov, and Zazzarro, 2013). It was also established that other price-related elements of loan contracts such as arrangement fees had a positive and mutually reinforcing effect, thus firms that are charged high loan arrangement fees will also face higher loan interest rates (Cowling & Yang, 2024).

However, as discussed earlier, there is a lack of understanding of the precise nature of the interest rates applied (either being fixed or variable), which could be well determined by both borrower-side and lender-side factors due to their own perceptions of future interest rate change and economic states. We consider this to be an important research gap as the financial consequences are not trivial between the fixed and variable rate loan even over a three-year loan maturity window. For example, if two identical firms took out a £50,000, three-year loan in December 2021, one with a fixed-rate of base plus 2.0% and the second an identical variable rate loan then the second firm would have seen its total loan interest rate increase to 7.0% by mid-July of 2023. This would increase its annual capital and interest repayments by £1260.72 over and above that repaid by the fixed rate loan firm.

Reflecting on this fixed and variable loan interest outcomes during the period spanning from 2020 and the depths of the Covid-19 pandemic and 2023, the high inflation period, we empirically test for the relative shares of fixed and variable interest rate lending on UK firms’ lending to identify whether the firm or lender bore the greatest share of the risk in the ‘bad’ future state of the economy that prevailed as evidence from Berger et al. (2025) showed that banks protected firms with long-term relationships from credit shocks where they had the capacity to do so. Further, we can identify what types of firms were able to access fixed rate and variable rate loans and on what type of debt. Here, the observed outcome of the nature of the interest rate being issued is the result of general equilibrium. The argument is that being issued with variable interest rate, firms rather than the lenders are bearing the interest rate risk, while if the debt is under fixed interest rate, the lenders are taking the interest rate risk.

Our baseline finding is that 19.45% of loans issued between 2020 and 2023 were issued on a variable interest rate and 80.55% on a fixed rate of interest. This suggests that in general, the lenders had taken most of the interest rate risk compared to borrowers. In terms of what types of firms accessed variable rate loans, we find that larger firms had a 10.79% higher percentage change in their probability and that firms that were willing to take investment risks were also more likely to be offered variable rate loans. For each one-unit shift on the risk tolerance scale, the percentage change in the percentage change in the probability of being issued a variable rate loan increased by 2.28%. This suggests that for a firm that is considered at higher risk level that willing to take higher risk, the loan term outcome reflects borrowers taking most of the interest rate risk burden. On the lender side of the loan market, we find that variable rate lending is specifically connected to mortgage loans which have a 49.13% higher percentage change in the probability of being issued at a variable interest rate. In contrast, P2P loans have an 11.58% lower percentage change in the probability of being issued at a variable rate. This risk-variable rate lending relationship was also identified in a US agricultural loan study which found that in general, it was optimal for farmers to pay up to 1.5 percentage points above variable interest rates to use some fixed-rate debt. Further, at fixed interest rates over 1.5 points above variable rate debt, the latter is chosen except for more risk-averse farmers (Leatham & Baker, 1988).

But further analysis at the firm size class level revealed that tolerance of investment risk mainly impacted on micro-firm loans in the sense that a risk-tolerant micro firm, the smallest size class, would face a much higher probability of being issued a variable rate loan than their small and medium-sized peers. Thus, firm size is important in understanding nuances in the probability of debt being issued at a variable rate of interest. It was also apparent that when analysing loans issued for different types of debt, medium-sized firms with overdrafts, credit card and hire purchase faced a higher probability of being issued a variable rate loan than their small and micro firm peers. The general relationships between a firm’s risk tolerance and variable interest rates were different for different types of debt. Specifically, risk tolerance increases the percentage change in the probability of being issued a variable rate bank loan by 12.23%, mortgage by 11.96%, hire purchase debt by 4.43%, overdrafts by 4.60% and credit card debt by 3.23%, but had no effect on director loans.

As the UK economy remains in a high base rate and lending rate equilibrium, it is evident that risk-tolerant micro firms, particularly those undertaking mortgage borrowing, and using bank loans and lines of credit, will face the consequences of higher interest rates on their variable rate loans. It is an empirical question whether this will impact on their probability of being able to service their debt in the future or whether the high-interest rate period will result in much higher default rates. Although this shifting of interest rate risk to risk-tolerant micro firms is important, it is also the case that in general medium and large firms are not immune as they have the highest probability of being issued a variable rate loan.

The rest of the paper is set out as follows: In Sect. 2, we present a borrower-side theoretical model of the fixed-variable interest rate decision with a specific focus on risk tolerance. We then present our data and the core descriptive statistics in Sect. 3. In Sect. 4, we discuss our econometric modelling results for fixed and variable interest rates. We conclude in Sect. 5.

## The fixed-variable interest rate model

In the later empirical analysis, we only observed the loan nature outcome that results from a general equilibrium from both the borrower-side self-selection model and lender-side screening model. However, given that the data we use is at business-level focusing on their investment and financial decision-making, we build a borrower-side self-selection model in this sector to support our later empirical test. Here, we consider a firm that makes decisions in a single period horizon. The firm possesses a von Neumann-Morgenstern utility function, *u*(*π*), defined over its end-of-period profit, *π*. The firm is non-satiated and risk averse in that *u*^/^(*π*) > 0 and *u*^//^(*π*) < 0 for all *π* > 0.

At the beginning of the period, the firm chooses an amount of investment, *k* ≥ 0, to generate revenue, *f* (*k*), at the end of the period. The revenue function, *f* (*k*), is strictly increasing and concave such that *f* (0) = 0 and $f{\prime}(0)=\infty$(the Inada condition). The firm finances the investment, *k*, by borrowing from a bank via a variable rate loan of amount, *k*_*v*_, and a fixed rate loan of amount, *k*_*f*_. The interest rate of the variable rate loan is ${\widetilde{r}}_{v}$, that is tied to the uncertain market interest rate, $\widetilde{r}>0$, by a fixed mark-up, *m* ≥ 0, so that ${\widetilde{r}}_{v}=\widetilde{r}+m$, where a tilde (^∼^) indicates a random variable. The interest rate on the fixed rate loan is *r*_*f*_ > 0 that is deterministic. To ensure that the fixed rate loan does not dominate the variable rate loan, we assume that ${r}_{f}>E[\widetilde{r}]+m$ so the fixed rate loan is expected to be more costly than the variable rate loan, where E[·] is the expectation operator with respect to the probability distribution of $\widetilde{r}$.

The firm’s end-of-period profit is given by

1 $$\widetilde{\pi }= f ({k}_{v}+ {k}_{f} ) - (1 + \widetilde{r} + m){k}_{v} - (1 +{r}_{f} ){k}_{f} + w$$

where *w* ≥ 0 is the known income from other operations and serves as a proxy for the size of the firm. The firm’s ex ante decision problem is to choose the amounts of the variable rate loan and that of the fixed rate loan, *k*_*v*_ ≥ 0 and *k*_*f*_ ≥ 0, so as to maximize the expected utility of its end-of-period profit:

2 $$E[u(\widetilde{\pi })]$$

where $\widetilde{\pi }$ is given by Eq. (1). The first-order conditions for program (2) are given by

3 $$E[u^{\prime}({\widetilde{\pi }}^{*})[f^{\prime}({k}^{*}) - (1 + \widetilde{r} + m)]]=0$$

and

4 $$E[u^{\prime}({\widetilde{\pi }}^{*})[f^{\prime}({k}^{*}) - (1 + {r}_{f})]]=0$$

where an asterisk (*) signifies an optimal level. The second-order conditions for program (2) are satisfied given the assumed properties of u(π) and f(k).

Since $u^{\prime}(\pi )>0$ for all π>0, Eq. (4) reduces to

5 $${f}^{\prime}\left({k}^{*}\right)=1 + {r}_{f}$$

Equation (5) states that the optimal amount of investment, ${k}^{*}={k}_{v}^{*}+{k}_{f }^{*},$ is the one at which the known marginal revenue,$f^{\prime}({k}^{*})$, is equal to the known marginal cost, $1 + {r}_{f}$. Hence, ${k}^{*}$ depends neither on the firm’s risk attitude nor on the interest rate uncertainty. Such a separation result is driven by the fact that the firm is free to borrow via the fixed rate loan that serves as a hedge against the interest rate uncertainty.

Substituting Eq. (5) into Eq. (3) yields

6 $$E[u^{\prime}({\widetilde{\pi }}^{*})({r}_{f} - \widetilde{r} - m)]=0$$

where ${\widetilde{\pi }}^{*}=f({k}^{*})+({r}_{f} - \widetilde{r} - m){k}_{v}^{*} - (1 + {r}_{f}) {k}_{f}^{*}+ w$. Using the covariance operator, $Cov[\bullet ,\bullet ]$, with respect to probability distribution of $\widetilde{r}$, we can write Eq. (6) as

$$E[u^{\prime}({\widetilde{\pi }}^{*})]\{{r}_{f} -E[\widetilde{r}] - m\} -Cov[u^{\prime}({\widetilde{\pi }}^{*}),\widetilde{r}]=0$$

which is equivalent to

7 $${r}_{f}=E[\widetilde{r}] +m+\frac{Cov[u^{\prime}({\widetilde{\pi }}^{*}),\widetilde{r}]}{E[u^{\prime}({\widetilde{\pi }}^{*})]}$$

Since $\frac{\partial u^{\prime}({\widetilde{\pi }}^{*})}{\partial r}=- u^{\prime\prime}({\widetilde{\pi }}^{*}){k}_{v}^{*} >0$, we have $Cov[u^{\prime}({\widetilde{\pi }}^{*}),\widetilde{r}]>0$. The last term on the right-hand side of Eq. (7) captures the risk premium demanded by the firm to take on the variable rate loan, which is positive due to the firm’s risk aversion. Equation (7) states that the firm’s optimal amount of the variable rate loan, ${k}_{v}^{*}$, is the one that equates the interest rate of the fixed rate loan to the certainty equivalent interest rate of the variable rate loan.

To examine the effect of greater risk aversion on the optimal mix of fixed and variable rate loans, we replace the firm’s utility function by $v\left(\mathrm\pi\right)=g\left(u\left(\mathrm\pi\right)\right)$, where$g\left(\bullet\right)$  is a strictly increasing and concave function (Pratt, 1964). The first-order conditions for program (2) become

8 $$E\left[g^{\prime}(u({\widetilde{\pi }}^{\dagger}))u^{\prime}({\widetilde{\pi }}^{\dagger})[f^{\prime}({k}^{\dagger})-(1 + \widetilde{r} + m)]\right]=0$$

and

9 $$E\left[g^{\prime}(u({\widetilde{\pi }}^{\dagger}))u^{\prime}({\widetilde{\pi }}^{\dagger})[f^{\prime}({k}^{\dagger})-(1 +{r}_{f})]\right]=0$$

where a dagger (†) signifies an optimal level. Equation (9) reduces to $f^{\prime}({k}^{\dagger})=(1 +{r}_{f})$ so that ${k}^{\dagger}={k}^{*}$ from Eq. (5). Substitution Eq. (5) into Eq. (8) yields

10 $$E\left[g^{\prime}(u({\widetilde{\pi }}^{\dagger}))u^{\prime}({\widetilde{\pi }}^{\dagger})({r}_{f} - \widetilde{r} - m)\right]=0$$

where ${\widetilde{\pi }}^{\dagger}=f({k}^{*})+({r}_{f} - \widetilde{r} - m){{k}_{v}}^{\dagger} - (1 + {r}_{f}){k}^{*}+ w.$ Evaluating the left-hand side of Eq. (10) at ${k}_{v}={k}_{v}^{*}$ yields

$$E\left[{g}^{\prime}\left(u\left({\widetilde{\pi }}^{*}\right)\right){u}^{\prime}\left({\widetilde{\pi }}^{*}\right)\left({r}_{f}-\widetilde{r}-m\right)\right]=E\left[\left[{g}^{\prime}\left(u\left({\widetilde{\pi }}^{*}\right)\right)-{g}^{\prime}\left(u\left({\pi }^{\diamond }\right)\right)\right]{u}^{\prime}\left({\widetilde{\pi }}^{*}\right)\left({r}_{f}-\widetilde{r}-m\right)\right]<0$$

where ${\pi }^{\diamond }=f({k}^{*})-(1+{r}_{f}){k}^{*} +w$, the equality follows from Eq. (6), and the inequality follows from $\frac{\partial g^{\prime}(u({\pi }^{*}))}{\partial r}=- g^{\prime\prime}(u({\pi }^{*}))u^{\prime}({\pi }^{*}){k}_{v}^{*} >0$. Hence, it follows from Eq. (10) and the second-order conditions for program (2) that ${{k}_{v}}^{\dagger}<{k}_{v}^{*}$ and ${{k}_{f}}^{\dagger}<{k}_{f}^{*}$ such that ${{k}_{v}}^{\dagger}+{{k}_{f}}^{\dagger}={k}_{v}^{*}+{k}_{f}^{*}$. We as such establish the following proposition.

### Proposition 1

Greater risk aversion induces a risk-averse firm to rely less on a variable rate loan and more on a fixed rate loan.

An alternative way to state Proposition 1 is that a more risk-tolerant firm relies more on a variable rate loan and less on a fixed rate loan. The intuition for Proposition 1 is as follows. The firm, being more risk-tolerant, finds the variable rate loan more attractive compared to the fixed rate, thereby demanding a smaller risk premium of the variable rate loan. The firm as such borrows more via the variable rate loan and less via the fixed rate loan to equate the certainty equivalent marginal cost of the former to the marginal cost of the latter at the optimum.

We now examine the effect of firm size on the optimal mix of fixed and variable rate loans. To this end, we totally differentiate Eq. (6) with respect to *w* to yield

11 $$\frac{d{k}_{v}^{*} }{dw}=- \frac{E[u^{\prime\prime}({\widetilde{\pi }}^{*})\left({r}_{f}-\widetilde{r}-m\right)]}{E[u^{\prime\prime}({\widetilde{\pi }}^{*}){\left({r}_{f}-\widetilde{r}-m\right)}^{2}]}$$

where ${\widetilde{\pi }}^{*}=f({k}^{*})+({r}_{f} - \widetilde{r} - m){k}_{v}^{*} - (1 + {r}_{f}) {k}_{f}^{*}+ w$. Hence, the sign of $\frac{d{k}_{v}^{*} }{dw}$ is the same as that of the numerator on the right-hand side of Eq. (11). We say that the firm exhibits decreasing absolute risk aversion (DARA) if $-\frac{u{\prime}{\prime}({\pi}_{2})}{u{\prime}({\pi}_{2})}<-\frac{u{\prime}{\prime}({\pi}_{1})}{u{\prime}({\pi}_{1})}$ for all ${\pi}_{2}>{\pi}_{1}>0$. Hence, we have

$$E[u^{\prime\prime}({\widetilde{\pi }}^{*})\left({r}_{f}-\widetilde{r}-m\right)]=E\left[\left[\frac{u^{\prime\prime}({\widetilde{\pi }}^{*})}{u^{\prime}({\widetilde{\pi }}^{*})}-\frac{u^{\prime\prime}({\pi }^{\diamond })}{u^{\prime}({\pi }^{\diamond })}\right]u^{\prime}({\widetilde{\pi }}^{*})({r}_{f} - \widetilde{r} - m)\right]>0$$

where ${\pi }^{\diamond }=f({k}^{*}) - (1 + {r}_{f}) {k}_{f}^{*}+ w$, the equality follows from Eq. (6), and the inequality follows from DARA. We conclude from Eqs. (5) and (11) that $\frac{d{k}_{v}^{*} }{dw}>0$ and $\frac{d{k}_{f}^{*} }{dw}<0$ such that $\frac{d{k}^{*} }{dw}=0$.

### Proposition 2

A larger firm size induces a firm that exhibits DARA to rely more on a variable rate loan and less on a fixed rate loan.

The intuition for Proposition 2 is as follows. DARA implies that the firm with a larger firm size becomes more risk tolerant. From Proposition 1, the more risk tolerant firm as such borrows more via the variable rate loan and less via the fixed rate loan so as to equate the certainty equivalent marginal cost of the former to the marginal cost of the latter at the optimum.

Before leaving this section, three caveats are in order. First, it is worth pointing out that the results of Proposition 2 hinges on the assumption of DARA. Under the alternative assumption of constant absolute risk aversion (CARA) or that of increasing absolute risk aversion (IARA), it can be easily shown in an analogous manner that firm size does not matter in the former or has the opposite effects in the latter on the optimal choice between variable and fixed rate loans, as compared to the those under DARA. Hence, it is an empirical question to determine the appropriate risk preference characterization.

Second, collateral, which is an important instrument commonly used in loan contracts, is not included in the model. The rationale is that the firm in the model does not default on its loans, and thus, collateral will not be seized by the bank. In a more elaborate model wherein default is possible, collateral will play a vital role in loan contracting.

Third, the model is static and focuses on the demand-side aspect of loan decisions. A more comprehensive model in a dynamic setting that also takes the supply-side aspect of loan decisions into account is called for. Our model should be regarded as a starting point for understanding how firms deal with financial uncertainty when facing turbulent economic conditions.

## Data and descriptive statistics

The data is derived from a business investment and financial decision-making survey funded by the Bank of England and the UK Department for Business and Trade. The survey was conducted in 2023, and the data covers the period from 2020–2021 to 2022–2023. In total, 2886 UK firms were surveyed, and a weighting system was constructed from the known UK business population to ensure representativeness of the survey sample to the business population. The key variable is whether a firm has debt that has been issued on a fixed or variable rate of interest. It is important to note that our data is at the firm-level, and we do not have the loan-level data to identify the debt origination time. The variable/fixed interest rate question was asked if a participant selected “currently use” a certain type of debt financing. Unfortunately, we do not have detailed information of the origination time of each debt. The data show that 19.45% of firms have debt issued on a variable rate of interest.

In terms of types of debt that firms use^2^ (see Table 1), credit card debt is the most common, and 51.53% of firms are currently using it. Overdrafts (lines of credit) are used by 19.71% of firms, family loans by 4.66%, mortgages by 8.42%, hire purchase by 25.22%, bank loans by 7.96% and factoring and invoice discounting by 3.45% of firms. Director’s loans had high usage at 15.23% but P2P lending had relatively minor use at only 1.41% of firms.

**Table 1:** Variable rate lending and use of different finance types and sources

Type of debt	Debt with variable interest rate %		% Used	
	Mean	St Dev	Mean	St Dev
Overdraft	33.31	47.18	19.71	39.79
Mortgage	67.98	46.74	8.42	27.77
Credit card	18.85	39.12	51.53	49.99
Factoring and invoice discounting	18.49	38.96	3.45	18.25
Hire Purchase	23.81	42.62	25.22	43.43
Bank loan	52.76	50.02	7.96	27.09
Family loan	32.48	47.03	4.66	21.08
Director loan	26.32	44.09	15.23	35.94
P2P	20.13	40.54	1.41	11.82

In terms of the respective shares of variable rate lending across the nine different types of debt, we observe some large variations. For example, 67.98% of mortgage debt is issued on a variable rate basis, as is 52.76% of bank loans, 33.31% of overdrafts (lines of credit) and 32.48% of family loans. This is much higher than the 18.49% of variable rate lending for factoring and invoice discounting and 18.85% for credit card debt. Sources of debt with intermediate level shares of variable rate lending include director’s loans at 26.32%, hire purchase at 23.81%, director’s loans at 26.32% and P2P at 20.13%. In general, short-term forms of debt are less likely to be issued at a variable rate of interest than longer-term debt.

There are also some differences in relative tolerance of investment risks^3^ between fixed and variable rate borrowers. On this, we find that variable rate lending is associated with a higher tolerance of investment risk with a mean score on the 5-point Likert scale of 3.50 (std dev = 1.16) compared to 3.16 (std dev = 1.25) for firms with fixed rate loans. Thus, risk-tolerant firms are more likely to have variable rate loans on average. This suggests that firms that are more willing to take investment risks are also more likely to bear the burden of interest rate risk. This might be a result of the effective screening process that lenders are capable of identifying such firms and are able to shift interest rate risk to those firms. Although our measurement of the risk tolerance is not observed by the lenders, during the lender screening process, they will have a general risk assessment of the business which we believe is highly correlated with our risk tolerance measurement reflecting the general risk level of the firm.

Table 2 reports the base incidence of variable loan interest rates by firm characteristics. Here, we observe that micro firms (1–9 employees) have the lowest share of variable rate loans at 18.56% and medium-sized firms (50 + employees) the highest share at 24.99%. Thus, medium-sized firms bear a greater share of the interest rate risk. On firm age, we find a negative pattern in the incidence of variable rate lending with the youngest firms (0–3 years old) having a variable interest rate share of 23.65% and the oldest firms (20 + years) having the lowest shares at 18.28%. Such an outcome could be the result of lenders accepting a greater degree of interest rate risk from older firms and shifting risk onto younger firms.

**Table 2:** Firm characteristics and variable rate debt

	Has debt with variable interest rate (%)	
	Mean	St Dev
Size class		
Micro	18.56	38.89
Small	24.13	42.83
Medium	24.99	43.37
Age class		
0–3	23.65	42.82
4–10	24.66	43.20
11–20	19.16	39.39
> 20	18.28	38.67
Industry		
A – Agriculture, forestry and fishing	45.46	50.07
B, D, E – Mining and quarrying, utilities	1.88	14.18
C – Manufacturing	15.52	36.30
F – Construction	16.44	37.14
G – Wholesale and retail, repair of motor vehicles	16.56	37.23
H – Transportation and storage	19.48	39.80
I – Accommodation and food service activities	33.16	47.27
J – Information and communication	12.20	32.85
K – Financial and insurance activities	18.43	38.97
L – Real estate activities	23.02	42.47
M – Professional, scientific and technical services	19.51	39.72
N – Administrative and support services	13.90	34.85
P – Public administration and defence	44.96	60.93
Q – Education	6.60	24.94
R – Arts, entertainment and recreation	22.43	41.87
S – Other service activities	18.47	38.87

At the industry sector level, we observe some large variations in the incidence of variable rate lending. Industry sectors with a high incidence of variable rate lending include agriculture, forestry, and fishing where 45.46% of debt is variable interest rate and public administration where variable rate lending accounts for 44.96% of lending. This contrasts with low incidences in mining and quarrying at 1.88% and education at 6.60%, although we note that mining and quarrying are sectors with low representation in the UK business population and as our sample is proportional to the population, in our survey sample.^4^ This may reflect, to some degree, the long-term nature of new investments in mining and education and the need for a fixed and constant repayment schedule.

Overall, the descriptive statistics show that larger and younger firms are more likely to have variable interest rate debt, and that there is substantial industry sector variation. Importantly, lenders seem to have appropriate risk assessment of the firms that correlated with a firm’s relative tolerance of investment risk, which could enable lenders to react by shifting interest rate risk to the borrower by issuing variable rate loans. There is also significant variation across different sources of debt, and this is somewhat related to whether the debt is shorter term or longer term, and whether it is a softer form of debt such as a director’s loan.

## Results

We estimated a full model to begin which included our rich firm and debt characteristics as explanatory variables to predict the relative probability of a firm of a specific type raising debt for a specific purpose being issued fixed or variable rate debt. As the dependent variable is coded 0 if the firm is issued a fixed rate loan and 1 if the firm is issued a variable rate loan, we choose to estimate a probit model of the following form:

$$Pr\left(X\right)=\Phi \left(X\beta \right)$$

Φ is the standard normal cumulative distribution function for the probit model, X is a vector of a set of explanatory variables and β is the vector of coefficients of the control variables. For ease of interpretation, we report the marginal probabilities that a firm with a characteristic *X* will have a higher or lower probability of being issued a variable rate loan compared to a firm without that characteristic. We also estimate a series of additional models that separately consider the fixed–variable rate probability for each firm size class and for each specific source and type of debt.

### The fixed–variable rate debt decision

From Table 3 Model [1], which reports the full model, we observe that medium-sized firms have a 10.79% percentage point change in the probability of being issued debt at a variable rate than micro firms. Firm age does not seem to be a significant factor determining the kind of interest rate that is being applied to the firms. On balance, our firm size results suggest that lenders are seeking to protect micro (and small) firms from interest rate risk by offering them fixed rate loans. This is consistent with using soft lending to lock-in customers to extract surplus profits from them in the future. Across industry sectors, we find that firms in real estate and education face a lower percentage point change in the probability of being issued variable rate debt and the respective percentage point changes in probability are −10.29% and −12.66%.

**Table 3:** Fixed–variable rate debt decision

Dependent variables	Being issued variable rate debt			
	[1] Full Model	[2] Micro Firms	[3] Small Firms	[4] Medium Firms
*Size class (base: micro)*				
Small	0.0328 (0.0257)			
Medium	0.1079*** (0.0453)			
Log(Age)	− 0.0195 (0.0123)	− 0.0164 (0.0140)	− 0.0608** (0.0308)	− 0.0015 (0.0297)
Tolerance of Investment Risk Scale (1–5)	0.0228*** (0.0080)	0.0238*** (0.0091)	0.0372* (0.0193)	0.0316 (0.0201)
Collateral	0.0105 (0.0088)	0.0106 (0.0103)	0.0145 (0.0181)	− 0.0177 (0.0220)
*Type of debt*				
Overdraft	0.1332*** (0.0253)	0.1354*** (0.0286)	0.1368** (0.0576)	0.1641** (0.0833)
Mortgage	0.4913*** (0.0420)	0.5204*** (0.0518)	0.4646*** (0.0716)	0.3197*** (0.1139)
Credit card	0.0031 (0.0200)	0.0103 (0.0225)	− 0.0303 (0.0502)	− 0.0904 (0.0745)
Factoring and invoice discounting	− 0.0276 (0.0386)	− 0.0585 (0.0482)	0.0105 (0.0742)	0.0906 (0.0985)
Hire purchase	0.0091 (0.0211)	0.0097 (0.0249)	− 0.0250 (0.0437)	0.0552 (0.0497)
Bank loan	0.2130*** (0.0437)	0.2443*** (0.0573)	0.1327** (0.0680)	0.3765*** (0.0923)
Family loan	0.0082 (0.0398)	− 0.0162 (0.0422)	0.2164** (0.1092)	0.0662 (0.2320)
Director loan	0.0438* (0.0267)	0.0452 (0.0294)	− 0.0054 (0.0585)	− 0.0227 (0.1102)
P2P	− 0.1158*** (0.0245)	− 0.1341** (0.0214)	− 0.0734 (0.0664)	0.1610 (0.2319)
*Sector (base: agriculture, forestry and fishing)*				
C – Manufacturing	− 0.0751* (0.0361)	− 0.0869* (0.0388)	0.0771 (0.1332)	− 0.0567 (0.0961)
F – Construction	− 0.0532 (0.0415)	− 0.0502 (0.0480)	0.0139 (0.1112)	0.0345 (0.1315)
G – Wholesale and retail, repair of motor vehicles	− 0.0611 (0.0378)	− 0.0635 (0.0427)	0.0661 (0.1287)	− 0.0391 (0.1009)
H – Transportation and storage	− 0.0353 (0.0534)	− 0.0220 (0.0623)	− 0.1263 (0.0789)	− 0.1014 (0.0860)
I – Accommodation and food service activities	− 0.0493 (0.0459)	− 0.0591 (0.0499)	0.0626 (0.1388)	− 0.0664 (0.0933)
J – Information and communication	− 0.0620 (0.0422)	− 0.0617 (0.0476)	0.0880 (0.1568)	− 0.1143 (0.0680)
K – Financial and insurance activities	− 0.0220 (0.0537)	− 0.0108 (0.0623)	− 0.0834 (0.1043)	
L – Real estate activities	− 0.1029** (0.0349)	− 0.0526 (0.0533)	− 0.1994** (0.0320)	
M – Professional, scientific and technical services	− 0.0216 (0.0469)	− 0.0027 (0.0574)	− 0.0691 (0.0914)	− 0.0682 (0.0860)
N – Administrative and support services	− 0.0649 (0.0470)	− 0.0642 (0.0527)	0.0055 (0.1436)	0.0317 (0.1657)
Q – Education	− 0.1266*** (0.0254)		− 0.0619 (0.1204)	− 0.1508 (0.0959)
R – Arts, entertainment and recreation	− 0.0192 (0.0482)	− 0.0384 (0.0504)	0.2313 (0.1850)	0.0505 (0.1686)
S – Other service activities	− 0.0370 (0.0437)	− 0.0316 (0.0516)	0.0524 (0.1159)	− 0.1063 (0.0736)
Region control	Yes	Yes	Yes	Yes
No. observations	2,168	1,374	472	298
Significance	0.0000	0.0000	0.0000	0.0000
Pseudo R2	0.2342	0.2426	0.2730	0.3615

*** indicates significance at 1% level, ** indicates significance at 5% level and * indicates significance at 10% level. Figures in parentheses are standard errors. Sector B, D, E (mining and quarrying, utilities) and P (public administration and defence) dropped in the regression estimation in all the models

The results also show that firms’ risk tolerance is positively related to variable rate lending, and a one unit increase in the five-point risk scale is associated with a 2.28% increase in the percentage point probability of being issued debt on a variable rate. Thus, an otherwise similar firm apart from its relative preference for risk at both extremes of the risk scale would have a 9.12% difference in the percentage point probability of being issued a variable rate loan. In this respect, lenders seem able to transfer interest rate risk to borrowers with a higher risk tolerance and appetite. Interestingly, overall, having collateral or not does not make significant difference in the probability of being issued debt on a fixed or variable interest rate.

Across different types and sources of debt we find significant differences in the probability of debt being issued at a variable rate of interest. Specifically, we find that mortgage debts are associated with a 49.13% increase in the percentage point probability of being issued on a variable rate, bank loans a 21.30% increase, and lines of credit a 13.32% increase. This contrasts with P2P lending which has an 11.58% lower percentage point probability of being issued on a variable rate. These results show that the precise nature of debt and its source are important factors in the determination of whether debt is issued at a fixed or variable rate of interest. When the firm’s owner’s family lends money to the business, however, this has no impact on the type of interest rate offered. As mortgages were issued during the highly volatile and uncertain economic environment that prevailed in the UK from the pandemic period until now, lenders were seeking to transfer interest rate risk to borrowers.

### Firm size class and the fixed–variable interest rate decision

Models [2], [3] and [4] in Table 3 report our separate firm size class models for micro-, small- and medium-sized firms. Here, we find that firm age reduces the probability of debt being issued on a variable rate with a marginal effect of − 0.0608** for small firms but has no significant effects for micro and medium-sized firms. Thus, older small firms benefit from fixed rates of interest on their debt. We also find that small firms operating in the real estate sector have a 19.94% lower percentage point probability of being issued debt on a variable rate of interest.

Firm’s tolerance of risk was significantly associated with the probability of being issued debt on a variable interest rate for micro firms and marginally significant for small firms. However, no significant effects were found for medium-sized firms. This may suggest that lenders might be concerned about shifting interest rate risk to micro firm borrowers who have a relatively high tolerance for accepting risk, but much less concerned about, this issue if debt is issued to a small or medium-sized firm. As micro firms are the dominant firm size class, and more often associated with risk loving entrepreneurship, then this segment of the firm population is the most exposed to interest rate risk given the evolving state of the UK economy. Here, collateral still shows no significant effects in our separate firm size class models.

In terms of different types and sources of debt, we find significant differences in respect of the probability of debt being issued on a variable rate of interest. For lines of credit, we find that the percentage point change in the probability of variable interest rates increases from 13.54% for micro firms to 16.41% for medium-sized firms. For mortgages, the opposite is true as micro firms have a 52.04% increase in the percentage point change in the probability that their mortgage will be on a variable rate and medium-sized firms only a 31.97% increase in the percentage point change in the probability. In respect of bank loans, micro firms were found to face a 24.43% increase in the percentage point probability of being variable rate and this compares to a 37.65% increase for medium-sized firms. We also found that micro firms have a 13.41% lower percentage point probability of being issued P2P loans at a variable rate although no significant effects were observed for small or medium-sized firms. For family loans, only small firms have a 21.64% higher percentage point probability of being issued at a variable rate. Overall, it is apparent that different types and sources of debt treat firms of different size classes differently in respect of their fixed or variable interest rate decision. Micro firms bear the highest interest rate risk on mortgages and the lowest interest rate risk on P2P loans. Medium-sized firms bear the highest interest rate risk on lines of credit and bank loans.

### Types and sources of debt and the fixed–variable interest rate decision

Here, we report on the determination of fixed and variable interest rates for each different type and source of debt. The results are reported in Columns [1] to [6] in Table 4. We could not estimate models for factoring and invoice discounting, family loans or P2P due to limited numbers of observations. In respect of firm size classes, we find that medium-sized firms had a higher percentage point probability of being issued debt on a variable rate in the case of lines of credit, + 22.42%, credit card debt, + 14.28% and hire purchase, + 14.30%. This suggests that medium-sized firms have interest rate risk added when the debt they are using is short-term in its focus.

**Table 4:** Fixed–variable rate debt decision—types of debt

Dependent variables	Being issued variable rate debt					
	[1] Overdraft	[2] Mortgage	[3] Credit card	[4] Hire purchase	[5] Bank loan	[6] Director loan
*Size class (base: micro)*						
Small	0.0740 (0.0596)	− 0.0218 (0.0763)	0.0541** (0.0284)	0.0548 (0.0384)	− 0.1175 (0.0865)	0.0073 (0.0670)
Medium	0.2242** (0.0939)	− 0.0565 (0.1121)	0.1428*** (0.0427)	0.1430*** (0.0508)	0.1579 (0.1073)	0.0335 (0.1470)
Log (Age)	0.0223 (0.0332)	− 0.0466 (0.0500)	− 0.0114 (0.0154)	0.0064 (0.0230)	0.0270 (0.0499)	0.0367 (0.0298)
Tolerance of Investment Risk Scale (1–5)	0.0460** (0.0187)	0.1196*** (0.0316)	0.0323*** (0.0100)	0.0443*** (0.0149)	0.1223*** (0.0334)	0.0209 (0.0214)
Collateral	0.0426** (0.0197)	0.0073 (0.0318)	0.0238** (0.0105)	0.0180 (0.0166)	− 0.0668** (0.0328)	0.0389* (0.0208)
Sector control	Yes	Yes	Yes	Yes	Yes	Yes
No. observations	545	259	1,506	856	256	393
Significance	0.0000	0.0243	0.0000	0.0000	0.0004	0.0867
Pseudo R2	0.0867	0.1069	0.0515	0.0745	0.1663	0.0579

*** indicates significance at 1% level, ** indicates significance at 5% level and * indicates significance at 10% level. Figures in parentheses are standard errors

Firm’s tolerance of investment risk is also important in the fixed–variable interest rate decision, and here, we find that in all models where it is found to be significant, it acts to increase the percentage point probability that debt will be issued at a variable rate. For bank loans, the marginal effect is 0.1223***; for hire purchase, 0.0443***; for mortgages, it is 0.1196***; for lines of credit, 0.0460**; and for credit card debt, the marginal percentage point probability increases by 0.0323***. This strongly suggests that when multiple types of lenders observe that a borrower firm is relatively risk-tolerant, they act by offering more variable rate loans to shift the interest rate risk onto the borrower. In this respect, if bad future states of the economy prevail, then the most risk-tolerant firms will face the interest rate consequences. The opposite, of course, is true if a good state of the economy prevails in the future. Here, despite the insignificant role of collateral found in the previous baseline models in Table 3, for specific types of debt, collateral is important in the overdraft, credit card and bank loan models of the determination of fixed or variable interest rate.

### Fixed–variable rate debt decision—robustness check

We also conducted some additional models that took account of potential differences in firms’ risk tolerance and its impact on other explanatory variables by separating out firms into low-risk tolerance (1/2 on the risk tolerance scale), risk neutral (3 on the risk tolerance scale), and high-risk tolerance (4/5 on the risk tolerance scale). This risk tolerance separation (Appendix Table 5 Models 2, 3 and 4) showed that the lenders’ reaction to specific types of debt was different in terms of the magnitude of influence on the fixed or variable interest rate decision and most pronounced for high-risk borrowers in respect of overdrafts, mortgage debt and bank loans.

We also augmented with a firm size–age interaction term to test whether larger and older (or smaller and younger) firms faced different responses from lenders in respect of being issued fixed or variable rate loans (Appendix Table 5). This roughly approximates the Berger and Udell (1998) financial life-cycle theory and reflects the reduced information asymmetry present when firms become larger and older. However, only small, old firms with low risk tolerance are found to have lower probability to have variable rate debt. There is no significant effect of the other interaction terms which suggests that lenders are more likely to treat size and age as independent variables in their decision-making and that they are not multiplicative. We also included the size–age interaction terms in separate models for each type of debt finance and found no significance (Appendix Table 6).

Finally, we formally tested whether the initial firm selection of a specific type of debt was important in the subsequent lenders’ decision regarding fixed or variable interest rates (Appendix Table 5 Model 5).^5^ This augmented set of models should reveal if firms select a specific type of debt because they had a prior preference for obtaining fixed (or variable) rate debt. Our results here suggest that this was not the case, and the predicted source of debt selection terms were not found to be significant for any type of debt. Rather, the lenders’ final decision is mainly related to firms’ risk tolerance and the presence of collateral.

## Conclusion

We set out a theoretical model of lenders fixed–variable interest rate decisions when issuing debt with a particular focus on the role of a firm’s risk tolerance. We then empirically tested the predictions of the theory against a UK SME data set covering a period from 2020 to 2023 when UK inflation and interest rates rose dramatically and unexpectedly over a sustained period. The focus of our empirical analysis was on how lenders reacted to firm’s risk tolerance, although we also considered wider issues relating to firm size classes and the specific types and sources of debt. This is important, as debt issued on a variable interest rate basis exposes the firm to future adverse states of the economy whereas debt issued on a fixed rate basis offers some protection against this. Of course, if the economy moves into a low inflation and low-interest rate state in the future, then variable rate borrowers will benefit and fixed rate borrowers will be penalised.

Our first evidence shows that only 19.45% of firms had debt issued on a variable interest basis. In this sense, the majority of UK firms holding debt currently have some protection against, what became, the high inflation–high-interest rate period from 14th December 2021 onwards and reached a peak when Bank of England base rates were held at 5.25% between 10th August 2023 and 30th July 2024, from a low of 0.1% on the 23rd March 2020 at the onset of the Covid-19 pandemic in the UK. However, having nearly one in every five debt holding UK firms exposed to this magnitude of interest rate risk is an issue of concern and their debt repayments would have risen significantly over this period. We also note that on the 1 st of August 2022 the Bank of England’s Financial Policy Committee removed the mandatory mortgage stress test requirement. The stress test required checks on whether a borrower could manage their mortgage interest rate repayments if interest rates rose by around 3% above the standard rate.

Our findings relating to the effect of firms' risk tolerance show quite clearly that most forms of debt issued to risk-tolerant firms have a significantly higher probability of being issued at a variable rate of interest. This is largely concentrated amongst micro firm debt holders and most evident for bank loans, hire purchase and mortgage debt. However, medium-sized firms are not immune from being exposed to interest rate risk, and in general, they have a higher probability of being issued debt on a variable rate basis, although this is most concentrated on short-term forms of debt such as lines of credit and hire purchase agreements.

In terms of our theoretical model, which emphasised the importance of firms’ tolerance of risk, we find empirical evidence that is supportive of the central role that risk plays in the lender’s decision to offer debt at a variable rate of interest thus shifting interest rate risk to the borrower. In this respect, given the known unfolding of UK economic events since 2021, this unfavourable outturn will have resulted in many, and particularly micro, firms being exposed to interest rate risk and more expensive debt. If their current free cash generation cannot support this increase in the costs of their existing debts, then this may lead to a substantial increase in default risk.

Overall, our core contribution engages with the debates on small business financing and the terms on which it is offered, if it is offered at all. But we also engage with wider debates and make important contributions to entrepreneurship research in the context of alternative forms of entrepreneurial finance as small firms are increasingly seeking to raise capital from non-traditional sources (Block et al., 2018). For example, we found that micro firms and high-risk tolerant firms using P2P as their preferred source of debt were more likely to be offered fixed rate loans. This is important as fixed rate loans offer some protection against adverse states of the economy; thus, P2P borrowers are in a better position if there is an economic downturn than their mainstream traditional bank borrowing peers. We also engage with other debates relating to the supply-side of capital markets and how monetary policy might impact on debt institutions lending practices through the interest rate channel (Bellavitis et al., 2023) as lenders may transition to variable loan interest rates if monetary policy acts to raise the base interest rate, leaving only preferred borrowers protected by fixed interest rates.

Given that our data relates to the UK debt market at a unique moment in time after the severe Covid-19 pandemic period, it is fair to question whether the findings could be generalised to other contexts outside of the UK. We take the view that the general findings relating to collateral and risk tolerance might easily transcend country boundaries and are imminently testable. Equally, our findings around alternative finance option could easily transfer into other institutional contexts. Indeed, the nuance would be in the nature of the capital markets and country-specific differences, and this would provide an interesting basis for future research.

## Data Availability

The data is the property of the UK government and the authors do not have permission to share it.

## Appendix

**Table 5:** Fixed–variable rate debt decision—robustness check

Dependent variables	Being issued variable rate debt					
	[1] Full model	[2] Low-risk tolerance firms	[3] Medium-risk tolerance firms	[4] High-risk tolerance firms	[5] Loan selection control	[6] With Covid-19 gov scheme loan
*Size class (base: micro)*						
Small	0.1602 (0.1363)	0.4833** (0.2414)	-0.0529 (0.0284)	-0.0387 (0.1265)	0.4485 (0.4886)	0.0325 (0.0257)
Medium	-0.0294 (0.1043)	-0.0974 (0.0545)	0.2962 (0.4990)	0.0000 (0.1829)	0.7346 (0.4037)	0.1134*** (0.0459)
Log (Age)	-0.0166 (0.0138)	0.0047 (0.0200)	-0.0541*** (0.0185)	-0.0072 (0.0205)	-0.0090 (0.0894)	-0.0214* (0.0123)
Small * log (Age)	-0.0338 (0.0313)	-0.0901** (0.0413)	0.0552 (0.0382)	0.0188 (0.0445)		
Medium * log (Age)	0.0393 (0.0368)	0.0804 (0.0617)	0.0137 (0.0377)	0.0237 (0.0574)		
Tolerance of Investment Risk Scale (1–5)	0.0230*** (0.0080)				0.0356*** (0.0086)	0.0227*** (0.0080)
Collateral	0.0101 (0.0088)	-0.0043 (0.0136)	-0.0060 (0.0101)	0.0269** (0.0126)	0.0202** (0.0094)	0.0089 (0.0087)
*Type of debt*					***With 1 st stage loan selection***	
Overdraft	0.1333*** (0.0253)	0.1114*** (0.0398)	0.1254*** (0.0513)	0.1338*** (0.0365)	0.8449 (1.7444)	0.1237*** (0.0254)
Mortgage	0.4915*** (0.0420)	0.3477*** (0.0791)	0.4216*** (0.1288)	0.5617*** (0.0527)	− 0.5569 (0.8103)	0.4886*** (0.0422)
Credit card	0.0032 (0.0200)	0.0059 (0.0286)	− 0.0114 (0.0224)	0.0039 (0.0300)	− 0.2666 (1.3235)	0.0074 (0.0197)
Factoring and invoice discounting	− 0.0299 (0.0381)	− 0.0171 (0.0556)	− 0.0021 (0.0322)	− 0.0318 (0.0578)		− 0.0356 (0.0371)
Hire purchase	0.0097 (0.0211)	0.0344 (0.0323)	− 0.0378* (0.0199)	0.0198 (0.0311)	− 0.0987 (0.5288)	0.0045 (0.0210)
Bank loan	0.2126*** (0.0437)	0.1407** (0.0730)	0.1428** (0.0918)	0.2539*** (0.0606)	− 0.4673 (0.8587)	0.2090*** (0.0433)
Family loan	0.0086 (0.0399)	− 0.0756* (0.0281)	0.1553* (0.1440)	0.0877 (0.0657)	− 0.8482 (0.7089)	0.0044 (0.0398)
Director loan	0.0427* (0.0265)	0.1506*** (0.0548)	− 0.0045 (0.0282)	0.0058 (0.0352)	− 1.0940 (1.6266)	0.0416 (0.0266)
P2P	− 0.1162*** (0.0242)	− 0.0878 (0.0329)		− 0.1203** (0.0379)		− 0.1193*** (0.0226)
Covid-19 gov scheme loans						0.0524*** (0.0189)
Region control	Yes	Yes	Yes	Yes	Yes	Yes
Sector control	Yes	Yes	Yes	Yes	Yes	Yes
No. observations	2,168	665	299	1192	1,887	2,167
Significance	0.0000	0.0000	0.0000	0.0000	0.000	0.0000
Pseudo R2	0.2350	0.1961	0.5050	0.2736	0.0689	0.2384

*** indicates significance at 1% level, ** indicates significance at 5% level and * indicates significance at 10% level. Figures in parentheses are standard errors

**Table 6:** Fixed–variable rate debt decision—types of debt

Dependent variables	Being issued variable rate debt					
	[1] Overdraft	[2] Mortgage	[3] credit card	[4] Hire purchase	[5] Bank loan	[6] Director loan
*Size class (base: micro)*						
Small	0.2246 (0.2801)	0.1550 (0.2611)	0.2040 (0.1563)	0.2771 (0.2090)	0.1949 (0.2989)	0.2043 (0.2772)
Medium	0.0863 (0.3434)	− 0.5739 (0.3543)	− 0.0452 (0.1143)	− 0.0640 (0.1523)	0.3211 (0.2129)	− 0.2565 (0.0670)
Log(Age)	0.0266 (0.0372)	− 0.0434 (0.0563)	− 0.0088 (0.0177)	0.0123 (0.0285)	0.0550 (0.0600)	0.0454 (0.0326)
Small * log(Age)	− 0.0450 (0.0828)	− 0.0628 (0.1085)	− 0.0388 (0.0363)	− 0.0610 (0.0543)	− 0.1077 (0.1070)	− 0.0608 (0.0746)
Medium * log(Age)	0.0384 (0.1038)	0.1581 (0.1575)	0.0538 (0.0423)	0.0635 (0.0565)	− 0.0687 (0.1108)	0.1875 (0.2464)
Tolerance of Investment Risk Scale (1–5)	0.0460** (0.0187)	0.1181*** (0.0317)	0.0323*** (0.0099)	0.0436*** (0.0149)	0.1208*** (0.0338)	0.0216 (0.0214)
Collateral	0.0422** (0.0198)	0.0066 (0.0318)	0.0237** (0.0105)	0.0181 (0.0167)	− 0.0669** (0.0328)	0.0386* (0.0209)
Sector control	Yes	Yes	Yes	Yes	Yes	Yes
No. observations	545	259	1506	856	256	393
Significance	0.0000	0.0383	0.0000	0.0000	0.0004	0.1210
Pseudo R2	0.0871	0.1095	0.0527	0.0771	0.1699	0.0595

*** indicates significance at 1% level, ** indicates significance at 5% level and * indicates significance at 10% level. Figures in parentheses are standard errors

## Footnotes

1 Figures from UK Finance, June 2023.

2 There is another type of debt in the survey question selection option: Covid-19 government scheme loan. However, the survey captured all scheme loans (BBLS, CBILS, CLBILS etc.) in one selection of the survey question, while bounce back loans are all fixed rate interest rate loans. Therefore, we do not include this type of debt in our later estimation when controlling for types of debt. However, we were able to create a dummy variable indicator which was coded 1 if a firm had a Covid-19 government loan and 0 otherwise. The descriptive statistic shows that about 45.7% firm in the survey sample has Covid-19 government loans. When included in the main aggregate interest rate type equation as additional robustness check, it was found to be positive and significant (Appendix Table 5, model 6).

3 The risk tolerance measurement is from the survey Likert measure of the following statement “I am willing to take risks when considering investments made by the business.” It is worded in a way that measures the risk tolerance for financial investment rather than loan origination. From the borrower-side decision making, this intends to measure risk tolerance before investment given by firm’s current financial status.

4 In the achieved survey sample, there are 17 mining and quarrying firms, and once the population weight is applied, this is 10.17.

5 For the 1 st stage loan selection, we estimated a set of probit models with dependent dummy variables indicating whether firm use a particular type of debt and a vector of independent variables including firm characteristics. Then, we estimate the predicted value of the use of the specific type of debt in the estimation of debt being issued with variable interest rate in Appendix Table 5 model 5.
